# To admit mistakes is not a weakness: it is the first step on the road of progress

**DOI:** 10.1093/eurpub/ckad208

**Published:** 2024-02-05

**Authors:** Vasiliy Vlassov

**Affiliations:** Department of Health Care Administration and Economics, National Research University Higher School of Economics, Moscow, Russian Federation

While artillery roars at the East of Europe and Middle East, hunger in Africa is at the doorstep, the COVID-19 pandemic is stepping back on the scale of global burden. But does it mean that world may close this case as next pandemic probably will not arrive tomorrow? No one serious epidemiologist would agree with that. What we should do? To continue the line of preparedness as it was drawn since 2005? It is not enough.

Let’s limit to the big questions. We have to be critical about science and scientific advice. For example, the special issue of the ‘Health Policy’ journal is completely devoted to the lessons learned from the pandemic. One hardly finds any mention of the mistaken policy in the articles. Theme issues of other public health journals are similar. In my humble opinion at least some of the described not so bad outcomes are not the results of the effective conscious actions, but the results of the natural course of events which was not harmed enough by human’s wrong actions. It is what The Lancet commission called ‘failures’.^1^

International collaboration in the production of the drugs and devices had relied on the idea that during the epidemic the international efforts would help to solve the local problems. In reality, the international collaboration from the personal protective equipment (PPE) production to the vaccine distribution failed on the large scale. If there would be sizable PPE production in Europe and North America, the deficit of PPE during first months may be less dramatic.

The progress of the health systems building since World War 2 was severely influenced by the business management ideas. Practice of the lean led to the reduction of the hospital capacities, reduction of the reserves. As a result, the short overload exhausted the resources of the systems. Would the health care systems more old-style, with some reserves of beds, PPE and drugs, the problems may be less traumatic.

The World Health Assembly agreed on preparedness for the next pandemic.^2^ It appears that delegates presume that the content and the scale of the necessary preparedness is more or less clear. It is not. Since 2005 megatons of oseltamivir were stored and egg-based vaccine production was supported, and both appeared useless. This pandemic is of respiratory infection, but all elements designed to work against the new influenza strain were irrelevant.

It is a shame that many countries did literally nothing for epidemic preparedness during the years since 2005. Part of the responsibility is on us, epidemiologists—we did not push the research agenda of preparedness, did not find and highlight the absence of evidence for the potentially important interventions, like face masks and distancing, did not demand the appropriate resources allocation.

During first weeks of the pandemic there was a question—is this new coronavirus really new, or is it just one of the variants circulated before, and became ‘visible’ only because Chinese started to test for it? To our shame we learned that world has no systematic surveillance of the ‘respiratory’ viruses circulating. It had happened that world built the system of monitoring influenza viruses only. It was very narrow vision of the potential dangers.

The shock and awe of the first months of the pandemic excuse many mistakes of the time and makes excusable the exaggerated response in some countries during the initial period of the pandemic. Even disinfection of the streets, despite it was obviously useless from the first day. What is not excusable—the convulsions of repeated lockdowns and painful enforcement of the measures of control in some countries even in 2022, when their neighbors exercised small scale regulation of the public life with the similar success.

It is not excusable that many public health specialists approved use of vaccine certificates/passes when it was known that vaccination does not prevent infection and infectivity of vaccinated. This practice was used to press more people to accept the vaccine. Thus, the science was betrayed for the goal. The cost is not only the deep wound of the trust in science. In countries like Russia the ‘pandemic regime’ was used, and still in use to suppress the civil protests. Thousands were fined and imprisoned.

The current pandemic invites us to look again at the ecological studies. Last 60 years, there is the clear understanding that ecological studies are very weak in finding the causal relationships. It is put in every textbook. Anyway, the ecological research plagued the field. They are cheap and fast, and they have their legitimate place in hypothesis generation, but efforts to extract the evidence for action from the ecological studies were wrong.

The similar situation was with non-randomized studies of treatments. It was completely logical and legitimate to find which existing drugs may be effective against the new disease and use the pathophysiological reasoning for that. It is long existing knowledge that this reasoning is prone to mistakes, and only after the comparative trials the treatments should be introduced to the practice. Now we know from the subsequent randomized controlled trials (RCTs) that prone positioning is not much effective.^3^ But it was OK to try it and recommend for practice it before data from RCTs, because the possible harm was negligible. Very different was the idea to use aprotinin, potentially killing medicine. Aprotinin even had find the way to the national guidelines in some countries. Years continued the chain of horrors of mass use of untested drugs, like ivermectin, even after first RCTs find them ineffective. I tend to think that the road to the use of the drugs like ivermectin and hydroxychloroquine after non-comparative trials was built by the drug industry. Pharma promoted the ‘real world data’ for drugs evaluation and ridiculed the RCTs for long.

The harms of an epidemiological studies of weak design and non-comparative trials may be less profound, if medical journals kept their standards of publication. The proportion of papers retracted is unprecedented for COVID-19-related papers. The apotheosis was the publication of articles from the obviously fabricated study in major journals. There were number of reviewers and members of editorial boards still in office, who approved these publications to glide over the hype.

As professionals most of us had held cemented en masse on the early positions before sufficient evidence were accumulated. Most public health people suppressed the efforts of critical thinking on the positions held. In this sense we became entrapped in the emotional and counter intellectual crowd discourse up to vilification of opponents. This hard driving by professionals fed up the call by uneducated public to criminalize the mask non-wearing, putting un-vaccinated into reservations etc. It was not possible to prevent spread of new virus variants, but it was possible not to impose the ineffective restrictions, harmful for the life of the societies and the human rights.

This list of the mistakes and biases of the time of the current pandemic is not complete and not error-free. Author may be well wrong with some of them. It is the invitation to detect and discuss the mistakes. To detect mistakes is the first and necessary step to not to repeat them. To be brave to acknowledge the bias is the privilege of the science.
